# Supplementary material for: Enhanced nonlinear interaction of polaritons via excitonic Rydberg states in monolayer WSe2
Source: Nat Commun. 2021 Apr 15;12:2269. doi: 10.1038/s41467-021-22537-x (PMC8050076; doi:10.1038/s41467-021-22537-x)
Supplement: Supplementary file 1 — Supplementary Information [file 41467_2021_22537_MOESM1_ESM.pdf]

ENHANCED NONLINEAR INTERACTION OF POLARITONS VIA EXCITONIC RYDBERG STATES IN MONOLAYER WSe_2

Jie Gu[1,2], Valentin Walther[3], Lutz Waldecker[4], Daniel Rhodes[5], Archana Raja[6], James C. Hone[5], Tony F. Heinz[4,7],

Stéphane Kéna-Cohen[8], Thomas Pohl[3], Vinod M. Menon[1,2,*]

*Email: vmenon@ccny.cuny.edu

[1]Department of Physics, City College of New York, 160 Convent Ave., New York, NY 10031, USA.

[2]Department of Physics, Graduate Center of the City University of New York (CUNY), 365 Fifth Ave. New York, NY 10016, USA.

[3]Center for Complex Quantum systems, Department of Physics and Astronomy, Aarhus University, DK- 8000 Aarhus C, Denmark.

[4]Department of Applied Physics, Stanford University, Stanford, CA 94305, USA.

[5]Department of Mechanical Engineering, Columbia University, New York, NY 10027 USA.

[6]Molecular Foundry, Lawrence Berkeley National Laboratory, Berkeley, CA 94720, USA.

[7]SLAC National Accelerator Laboratory, Menlo Park, CA 94025, USA.

[8]Department of Engineering Physics, École Polytechnique de Montréal, Montréal, Quebec, Canada.

Supplementary Note 1. Sample fabrication

Details of the sample fabrication is shown in the method section. The cavity mode is controlled by spin coating different thicknesses of PMMA. Supplementary Fig. 1 shows two bare cavity reflection examples with different PMMA thicknesses. Both cavities have the same cavity structure (Top 40 nm Silver\ PMMA \ 12 periods bottom DBR). The cavity mode red shifts when the PMMA thickness is thicker.

The relevant area is that of the 2D material heterostructure, which in the present experiment was ~ 30 μm^2 for the 1s and 2s measurements. This is larger than the laser spot size of less than $1 \mu m^2$. Sample inhomogeneities are predominantly caused by the transfer process due to air bubbles being trapped, as shown in the Supplementary Fig. 2. However, the ~ 20 μm^2 area shown by the white circle was devoid of any such inhomogeneity and so using a $1 \mu m^2$ laser spot we can probe multiple locations on the sample where inhomogeneities are not an issue. Likewise, local strain induced inhomogeneities due to the stacked heterostructures are inherently present in these experiments but do not affect the overall conclusions of the measurements on the scale of the laser spot size.

Supplementary Note 2. Theoretical description of the nonlinearity

The cavity equation of motion of a coherent field $\mathcal{E}$ in a cavity is given by its coupling to an excitonic resonance, denoted by $\hat{X}$, with coupling constant g, its detuning with respect to the cavity resonance $\Delta_{\text{cav}} = \omega_{\text{in}} - \omega_{\text{cav}}$, decay from the cavity at a rate κ as well as a driving term E^{in} with coupling constant η [1]

$$\partial_t \mathcal{E}(\mathbf{r}) = -ig\hat{X}(\mathbf{r}) - \frac{\Gamma_{\text{cav}}}{2}\mathcal{E}(\mathbf{r}) + \eta E^{\text{in}}. \tag{1}$$

Here, we summarized detuning and decay into $\Gamma_{\text{cav}} = \kappa - 2i\Delta_{\text{cav}}$. Note that the detuning contains the dispersion of the optical cavity field and can therefore be tuned by changing the incident angle of the external driving field in our experiment. The excitonic dynamics, in turn, are given by

$$\partial_t \hat{X}(\mathbf{r}) = -ig\mathcal{E}(\mathbf{r}) - \frac{\Gamma}{2}\hat{X}(\mathbf{r}) - i\int d\mathbf{r}' V(|\mathbf{r}-\mathbf{r}'|)\hat{X}^\dagger(\mathbf{r}')\hat{X}(\mathbf{r}')\hat{X}(\mathbf{r}). \tag{2}$$

for a general exciton-exciton interaction potential $V(\mathbf{r}-\mathbf{r}')$ and with excitonic loss channel γ and detuning with respect to the exciton $\Delta = \omega_{\text{in}} - \omega_X$, summarized in $\Gamma = \gamma - 2i\Delta$. Reflection is proportional to the cavity steady-state field intensity $|\mathcal{E}|^2$. It is important to note that Eq. (2) cannot be solved perturbatively in the interaction V which typically is very strong at short distances. Instead, a solution is sought for small driving fields, corresponding to a cluster expansion in the exciton correlations, as outlined in [2, 3]. Since the leading order nonlinearity is determined by terms up to pair correlations, the hierarchy can be truncated at this order. The required set of equations of motion reads

$$\frac{\partial}{\partial t}\hat{X}^\dagger(\mathbf{r}')\hat{X}(\mathbf{r}) = -ig\mathcal{E}(\mathbf{r})\hat{X}^\dagger(\mathbf{r}') + ig\mathcal{E}^*(\mathbf{r}')\hat{X}(\mathbf{r}) - \left(\frac{\Gamma}{2}+\frac{\Gamma^*}{2}\right)\hat{X}^\dagger(\mathbf{r}')\hat{X}(\mathbf{r}). \tag{3}$$

$$\frac{\partial}{\partial t}\hat{X}(\mathbf{r})\hat{X}(\mathbf{r}') = -ig\mathcal{E}(\mathbf{r})\hat{X}(\mathbf{r}') - ig\mathcal{E}(\mathbf{r}')\hat{X}(\mathbf{r}) - \Gamma\hat{X}(\mathbf{r})\hat{X}(\mathbf{r}') - iV(|\mathbf{r}-\mathbf{r}'|)\hat{X}(\mathbf{r})\hat{X}(\mathbf{r}'). \tag{4}$$

$$\frac{\partial}{\partial t}\hat{X}^\dagger(\mathbf{r})\hat{X}(\mathbf{r}) = -ig\mathcal{E}(\mathbf{r})\hat{X}^\dagger(\mathbf{r}) + ig\mathcal{E}^*(\mathbf{r})\hat{X}(\mathbf{r}) - \left(\frac{\Gamma^*}{2}+\frac{\Gamma}{2}\right)\hat{X}^\dagger(\mathbf{r})\hat{X}(\mathbf{r}). \tag{5}$$

$$\frac{\partial}{\partial t}\hat{X}^\dagger(\mathbf{r})\hat{X}(\mathbf{r})\hat{X}(\mathbf{r}') = -ig\mathcal{E}(\mathbf{r})\hat{X}^\dagger(\mathbf{r})\hat{X}(\mathbf{r}') + ig\mathcal{E}^*(\mathbf{r})\hat{X}(\mathbf{r})\hat{X}(\mathbf{r}') - ig\mathcal{E}(\mathbf{r}')\hat{X}^\dagger(\mathbf{r})\hat{X}(\mathbf{r}) - \left(\frac{\Gamma^*}{2}+\Gamma+iV(|\mathbf{r}-\mathbf{r}'|)\right)\hat{X}^\dagger(\mathbf{r})\hat{X}(\mathbf{r})\hat{X}(\mathbf{r}'). \tag{6}$$

Considering steady-state expectation values, a closed algebraic system of equations is obtained that can be solved in an expansion in $\mathcal{E}$. Upon insertion of the solution $\langle\hat{X}(\mathbf{r})\rangle$ into Eq. (1), a third-order nonlinear cavity field equation emerges

$$\partial_t\mathcal{E}(\mathbf{r}) = \chi^{(1)}\mathcal{E}(\mathbf{r}) + \int d\mathbf{r}'\chi^{(3)}(\mathbf{r}-\mathbf{r}')|\mathcal{E}(\mathbf{r}')|^2\mathcal{E}(\mathbf{r}) - \frac{\Gamma_{\text{cav}}}{2}\mathcal{E}(\mathbf{r}) + \eta E^{\text{in}}. \tag{7}$$

with the linear (nonlinear) susceptibility $\chi^{(1)}$ ($\chi^{(3)}$)

$$\chi^{(1)} = -\frac{2g^2}{\Gamma}. \tag{8}$$

$$\chi^{(3)}(\mathbf{r}) = \frac{16g^4}{|\Gamma|^2\Gamma}\frac{iU(\mathbf{r})}{\Gamma+iU(\mathbf{r})},. \tag{9}$$

as given in Eq. (1) in the main text. The nonlocal character of the nonlinear susceptibility reflects the finite exciton-exciton interaction range and the ensuing blockade effect at short exciton separations. Assuming a spatially flat cavity field, we define $\bar{\chi}^{(3)} = \int d\mathbf{r}\chi^{(3)}(\mathbf{r})$ to get a local third-order equation

$$\chi^{(1)}\mathcal{E} + \bar{\chi}^{(3)}|\mathcal{E}|^2\mathcal{E} - \frac{\Gamma_{\text{cav}}}{2}\mathcal{E} + \eta E^{\text{in}} = 0. \tag{10}$$

To obtain the first-order shift of the cavity resonance, it is illuminating to solve Eq. (10) perturbatively in the nonlinearity

$$\mathcal{E}^{(0)} = -\frac{\eta E^{\text{in}}}{\chi^{(1)} - \frac{\Gamma_{\text{cav}}}{2}} \tag{11}$$

$$\mathcal{E}^{(1)} = -\frac{\eta E^{\text{in}}}{\chi^{(1)} + \bar{\chi}^{(3)}|\mathcal{E}^{(0)}|^2 - \frac{\Gamma_{\text{cav}}}{2}}. \tag{12}$$

Eq. (11) is the linear limit, where exciton interaction are negligible. We evaluate the nonlinear part explicitly for a model potential $U(r) = U_0\Theta(r - R_{\text{bl}})$, and take the limit $U_0 \to \infty$ (hard-core bosons), yielding

$$\bar{\chi}^{(3)} = \frac{16g^4(\gamma + 2i\Delta)}{(\gamma^2 + 4\Delta^2)^2} \pi R_c^2. \tag{13}$$

Within this simple model capturing the short-ranged exciton repulsion, the imaginary part of the photonic nonlinearity changes sign with the detuning Δ. The maxima of the field $|\mathcal{E}^{(1)}|^2$ as a function of Δ are then given by the minima of the denominator of Eq. (12) and can, for $|\Delta| \approx g \gg \gamma$, approximately be determined from the root equation

$$x^2 - x + 2\pi R_{\mathrm{bl}}^2 |\mathcal{E}^{(0)}|^2 = 0 \tag{14}$$

with $x = (\Delta/g)^2$, which are at $\Delta \approx \pm g(1 - \pi R_{\mathrm{bl}}^2 |\mathcal{E}^{(0)}|^2)$. Defining the cavity Rabi splitting at zero field as $\Omega_0 = 2g$, the low-intensity cavity splitting Ω_n as a function of the polariton density is

$$\Omega_n = \Omega_0 \left(1 - \frac{\pi}{2} R_{\mathrm{bl}}^2 n\right), \tag{15}$$

as given in the main text.

Supplementary Note 3. Polariton density calculation

Polariton density $|\psi_P(\alpha, t)|^2$ at any given wavevector α in the momentum space, is calculated by the Gross-Pitaevskii equation of the form

$$i\hbar \frac{\partial \psi_{LP}(\alpha, t)}{\partial t} = \left[\epsilon_{LP}(\alpha) - \frac{i\hbar\gamma_{LP}}{2}\right] \psi_{LP}(\alpha, t) + \hbar F_P(\alpha, t) \tag{16}$$

The $\epsilon_{LP}(\alpha) = \hbar\omega_{LP}(\alpha)$ is the polariton dispersion of the lower branch and γ_{LP} is the polariton loss rate. In our experiment, γ_{LP} is $10.8 \times 10^{12}/s$. $F_P(\alpha, t)$ is the pumping term. The pumping pulse width is 20 ps, which is much larger than the polariton lifetime (94 fs), so the pumping term can be treated as a CW source that is in resonant with the polariton branch, with a harmonic form like $\hbar F_P(\alpha) e^{-i\omega_P t}$. We can define $\psi_{LP}(\alpha, t) = \tilde{\psi}_{LP}(\alpha) e^{-i\omega_P t}$, then the above equation can be rewritten as

$$\left[\hbar\omega_P - \epsilon_{LP}(\alpha) + \frac{i\hbar\gamma_{LP}}{2}\right] \tilde{\psi}_{LP}(\alpha) = \hbar F_P(\alpha) \tag{17}$$

The $F_P(\alpha)$ term is governed by the input-output relation[1]

$$F_P(\alpha) = C(\alpha) \sqrt{\frac{\kappa \cdot P_{int}(\alpha)}{\hbar\omega_P}} \tag{18}$$

κ was estimated accounting for the DBR penetration depth and phase change from the silver mirror.

$$\kappa = \frac{|t_{top\_mirror}|^2}{\tau_{trip}} \tag{19}$$

Here $C(\alpha)$ is the polariton branch photon Hopfield coefficient. $P_{int}(\alpha)$ is the incident power on the top surface and κ is the coupling coefficient, t_{top_mirror} is the transmission of top mirror, and τ_{trip} is the photon trip time in the cavity. This gives $\kappa = 4.3 \times 10^{12}\ rad/s$. Combining equation above, we have

$$\tilde{\psi}_{LP}(\alpha) = \frac{\hbar F_P(\alpha)}{\hbar\omega_P - \epsilon_{LP}(\alpha) + \frac{i\hbar\gamma_{LP}}{2}} \tag{20}$$

$$|\psi_{LP}(\alpha,t)|^2 = \left|\tilde{\psi}_{LP}(\alpha)\right|^2 = \frac{|C|^2 \frac{\kappa \cdot P_{int}(\alpha)}{\hbar\omega_P}}{(\omega_P - \omega_{LP}(\alpha))^2 + \left(\frac{\gamma_{LP}}{2}\right)^2} \tag{21}$$

The total density can be evaluated as an integral over the resonance region:

$$|\psi_{LP}(\alpha,t)|^2 = \int \frac{|C|^2 \frac{\kappa \cdot \xi(\alpha)}{\hbar\omega_P}}{(\omega_P - \omega_{LP}(\alpha))^2 + \left(\frac{\gamma_{LP}}{2}\right)^2} d\omega_P \tag{22}$$

where $\xi(\alpha)$ is the power density in the small resonance region, which is considered to be flat. So equation (22) can be estimated as an integral of a Lorentzian, it gives:

$$|\psi_{LP}(\alpha,t)|^2 = \frac{2\pi|C|^2\kappa \cdot \xi}{\hbar\omega_{LP}\gamma_{LP}} \tag{23}$$

We use the following to determine polariton loss:

$$\gamma_{LP}(\alpha) = |C_{LP}(\alpha)|^2\zeta + |X_{LP}(\alpha)|^2\gamma. \tag{24}$$

Here $C_{LP}(\alpha)$ and $X_{LP}(\alpha)$ denotes the photonic and excitonic Hopfield coefficient for the lower polariton, respectively. The constants ζ and γ are the decay rates of the cavity and the exciton, respectively. Both of our fabricated cavities for the 1s and 2s exciton reach a photon lifetime of ζ^{-1}= 94 fs. The excitons have a lifetime of approximately $\gamma^{-1} = 2ps$ for both the 1s exciton in WS_2 and the 2s exciton in WSe_2[5]. An analogous expression is evaluated for the upper polariton branch to obtain the upper polariton density $\rho_{UP}(\alpha)$. From the two densities, $\rho_{LP}(\alpha)$ and $\rho_{UP}(\alpha)$, we obtain the exciton density at given angle

$$\rho_X(\alpha) = |X_{LP}(\alpha)|^2\rho_{LP}(\alpha) + |X_{UP}(\alpha)|^2\rho_{UP}(\alpha), \tag{25}$$

using the excitonic Hopfield coefficients $X_{LP}(\alpha)$ and $X_{UP}(\alpha)$. Finally, we calculate the total exciton density by summing over all measured angles, $n_X = \sum_\alpha \rho_X(\alpha)$. Around the avoided crossing, the total polariton density is approximately twice the exciton density and can therefore be estimated as $n = 2n_X$. This value is used to obtain the density scale in Fig. 4b,c of the main text and to determine the blockade radius from the depicted data and Eq. (2) of the main text.

The above analysis requires a reliable determination of the different Hopfield coefficients, i.e. the eigenstate coefficients of the matrix

$$\begin{pmatrix} \Delta_{\text{cav}}(\alpha) - i\frac{\zeta}{2} & g \\ g & \Delta - i\frac{\gamma}{2} \end{pmatrix}$$

that yields coupled photon-exciton dynamics as described by Eq. (1) and (2) in the linear regime ($V = 0$), as discussed in the previous section. The free parameters are readily obtained by fitting the linear

transmission spectrum obtained from Eq. (1) and (2) to our low-intensity measurements for a given incident angle α. This approach yields a straightforward and accurate determination of the α -dependent (defining the in-plane momentum) Hopfield coefficients, and intrinsically accounts for any temperature dependencies and other experimental effects on the Hopfield coefficients.

Supplementary Figures

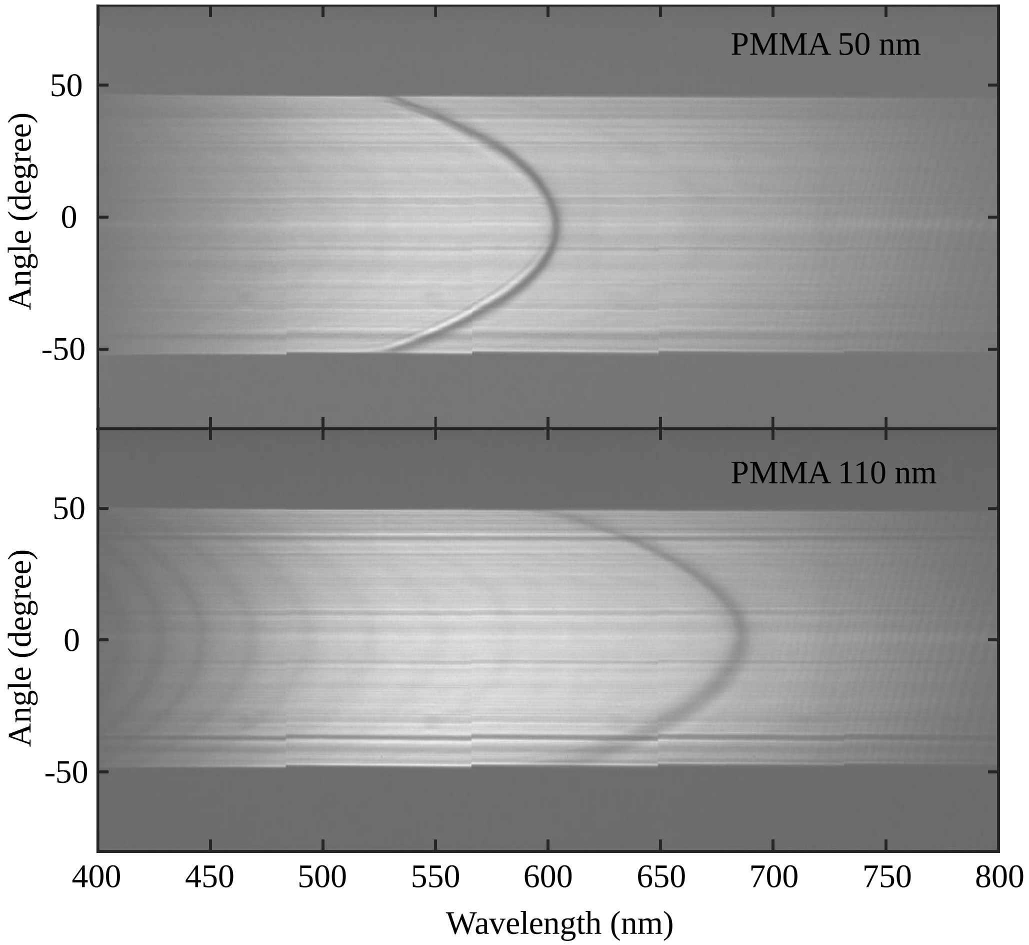

Supplementary Figure 1 | Bare cavity. Angle resolved reflection spectra of two bare cavities with different PMMA thickness at room temperature.

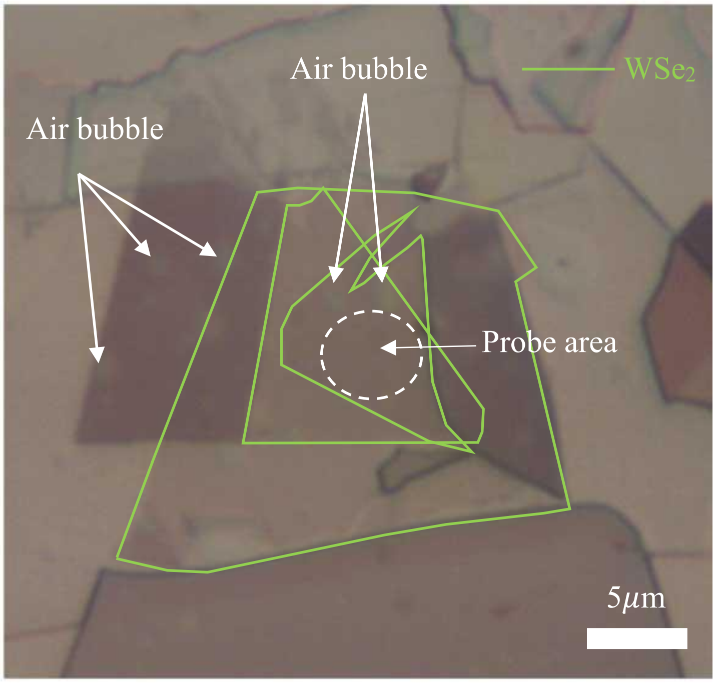

Supplementary Figure 2 | Optical image of stack. Optical image shows the stack on DBR surface. The green lines represent three WSe_2 monolayers' edges. White arrows show air bubbles originates from the stacking process.

a

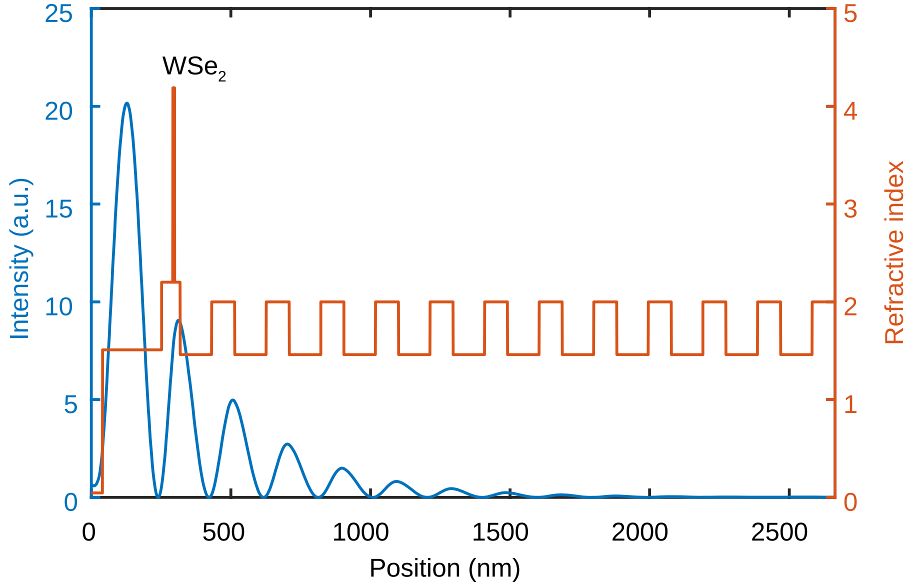

b

Material	Silver	PMMA	hBN	WSe_2	hBN	WSe_2	hBN	WSe_2	hBN	DBR-SiO_2	DBR-SiNx
Thickness (nm)	40	212	40	0.7	2	0.7	2	0.7	20	113	82.5

Supplementary Figure 3 | Field distribution of WSe_2 cavity. **a,** Transfer matrix simulation (blue curve) of the field distribution at 670nm for structure shown in Fig. 1a. Position '0' is related to the top silver surface. The sharp peak in the refractive index plot (orange curve) shows the relative position of WSe_2 in the cavity. **b,** Thickness of each layer for the WSe_2 cavity structure in Fig. 1a.

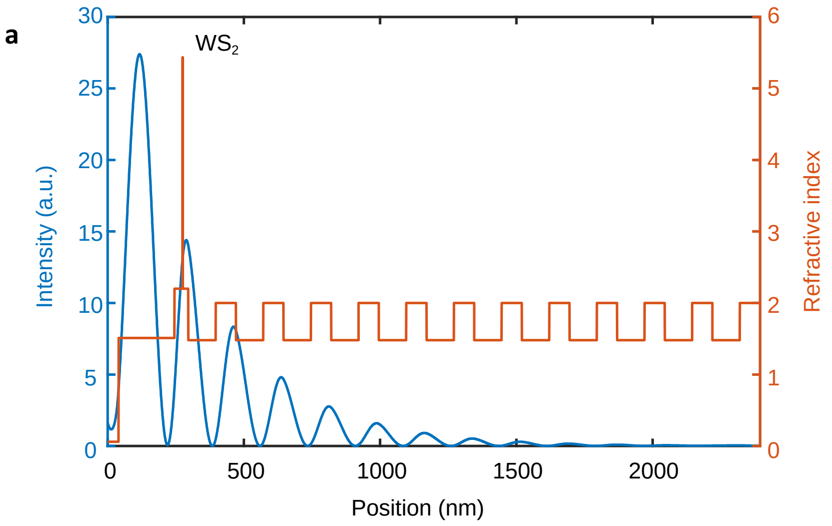

b

Material	Silver	PMMA	hBN	WS_2	hBN	DBR-SiO_2	DBR-SiNx
Thickness (nm)	40	205	30	0.7	20	102	73

Supplementary Figure 4 | Field distribution of WS_2 cavity. a, Transfer matrix simulation (blue curve) of the field distribution at 600nm for the WS2 cavity. Position '0' is related to the top silver surface. The sharp peak in the refractive index plot (orange curve) shows the relative position of WS_2 in the cavity. **b,** Thickness of each layer for the WS_2 cavity structure

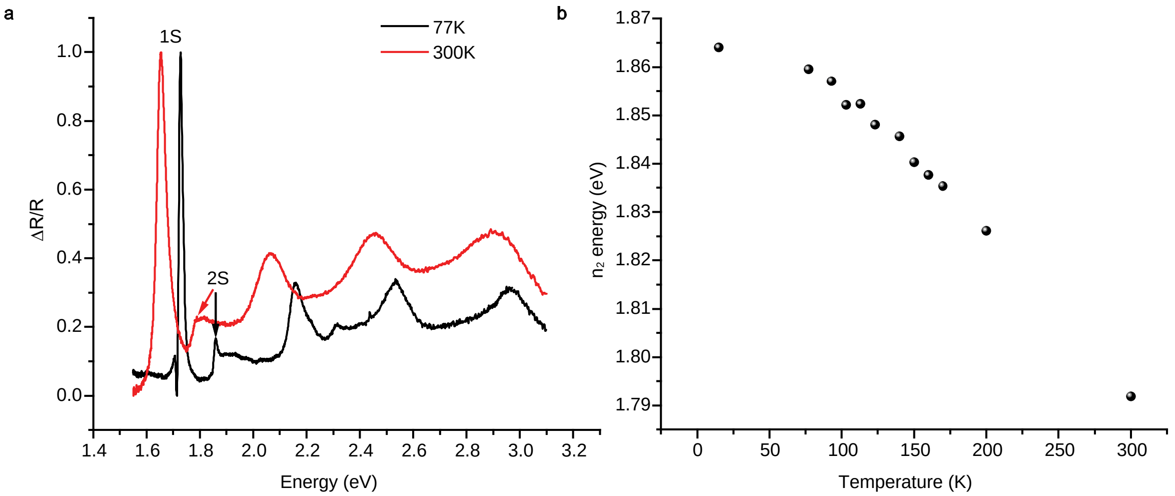

Supplementary Figure 5 | Temperature dependent 2s exciton energy. a, Normalized differential reflection spectra of monolayer WSe_2 at temperature of 77K (black) and 300K (red). Clear 2s peak was observed even at room temperature. The relative height of the 2s peak is 5-10 times lower than the main exciton peak (1s), indicating much smaller oscillator strength for the 2s state. **b**, 2s energy as a function of temperature. The blue shift from 300K to 15K is about 70 meV.

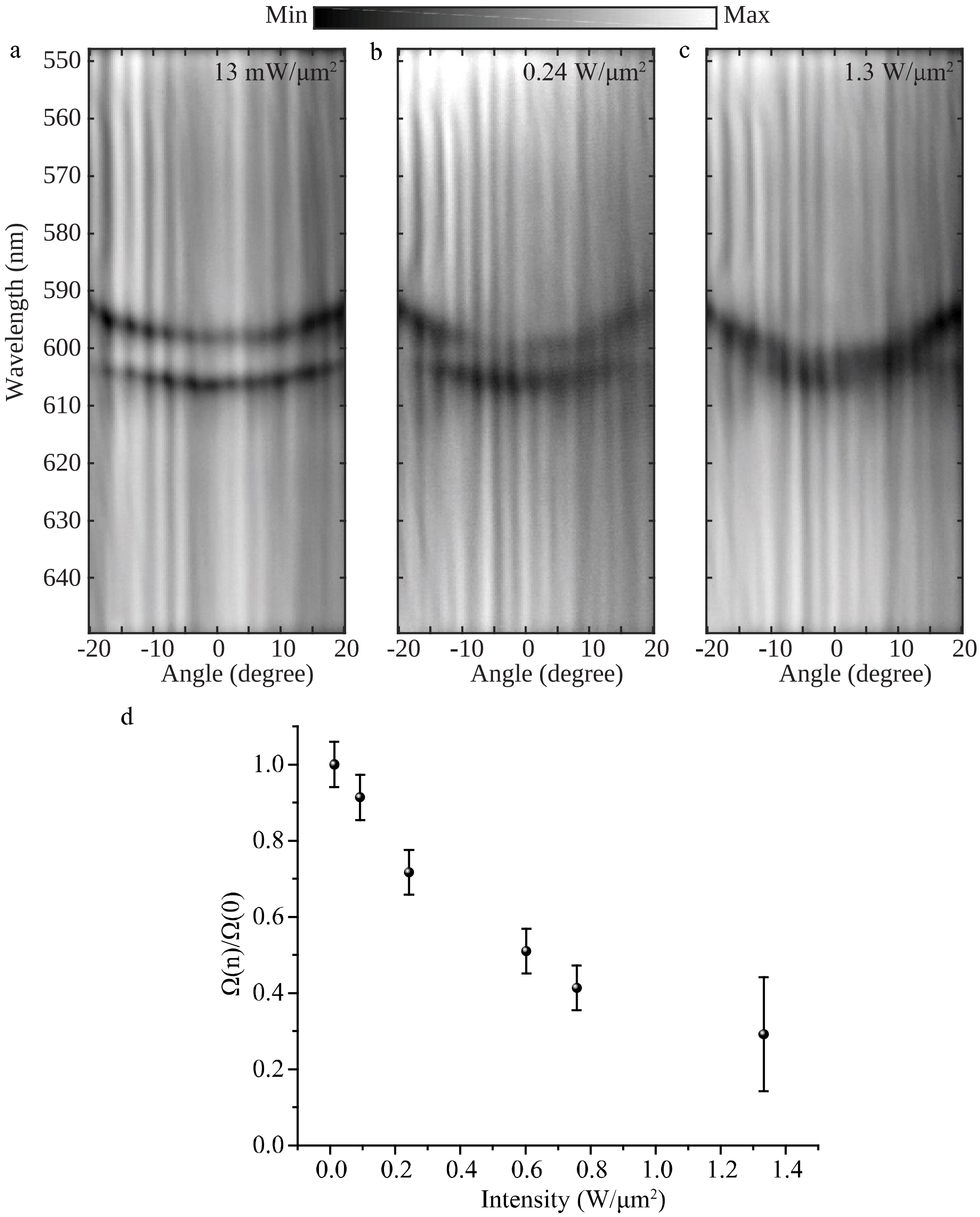

Supplementary Figure 6 | 1s exciton polariton intensity dependent Rabi splitting at 77K. a-c, White light reflection at the single pulse power of $13\ \mathrm{mW/\mu m^2}$ (**a**), $0.24\ \mathrm{W/\mu m^2}$ (**b**), $1.3\ \mathrm{W/\mu m^2}$ (**c**). As power is increasing, the Rabi splitting becomes smaller. **d**, Normalized Rabi splitting as a function of intensity. The errors bars represent one standard deviation from fitting the reflectivity at each input power.

Supplementary References

[1] I. Carusotto, & Chr. Ciuti, Rev. Mod. Phys. **85**, 299 (2013).

[2] S. Sevin ç çli, N. Henkel, C. Ates & T. Pohl, Phys. Rev. Lett. **107**, 153001 (2011).

[3] V. Walther, R. Johne & T. Pohl, Nat. Comm. **9**, 1309 (2018).

[4] F. Barachati et al, I. Nature Nanotech. **13**, 906-909 (2018).

[5] M. Palummo et al, Nano Lett. **15**, 2794-2800 (2015)
